# B‑Site Cu^2^^+^ Substitution and Strain-Mediated Magnetic Evolution in La_2_CoRuO_6_ Double Perovskite: Insights from Experiment and DFT + U‑Corrected Calculations

**DOI:** 10.1021/acsami.5c18178

**Published:** 2025-12-15

**Authors:** Sibusiso Nqayi, Buyisiwe Sondezi

**Affiliations:** Rare Earth-Based Oxides and Nano Group, Department of Physics, 61799 University of Johannesburg, Cnr Kingsway Avenue and University Road, Auckland Park, Johannesburg 2006, South Africa

**Keywords:** double perovskites, DFT + U corrections, modified Curie−Weiss (CW), B-site substitution, ferromagnetism (FM), antiferromagnetism (AFM)

## Abstract

Understanding the magnetic ground states of double perovskites remains complex due to competing exchange interactions, spin–orbit coupling, and structural disorder. This study explores the substitution of Cu^2^^+^ for Co^2^^+^ in La_2_CoRuO_6_ (LCRO), integrating experimental and DFT methods to probe the structural and electronic effects influencing magnetism. Pristine LCRO exhibits a monoclinic *P*2_1_/*c* phase with dominant antiferromagnetic (AFM) Co^2^^+^–O–Ru^4^^+^ interactions. Low-level Cu^2^^+^ substitution (*x* = 0.05 and 0.3) induces a strain-driven transformation to a tetragonal *I*4/*m* phase, introducing structural inhomogeneity and mixed valence states. These lead to competing ferromagnetic (FM) interactions (Cu^2^^+^–O–Ru^4^^+^/Cu^2^^+^), while AFM order partially persists at *x* = 0.3 due to orbital asymmetry and strain effects. Magnetic measurements and DFT calculations show a Néel temperature (*T*_N_) shift from 28.7 to 39.8 K (*x* = 0.05), and emerging FM behavior at 19.2 K. At *x* = 0.3, AFM suppression and a Curie temperature (*T*_C_) of 36.5 K reveal dominant FM pathways. Finite-size corrected Curie–Weiss analysis highlights the role of strain and particle size in modulating magnetic properties and restoring intrinsic behavior in larger particles.

## Graphical abstract

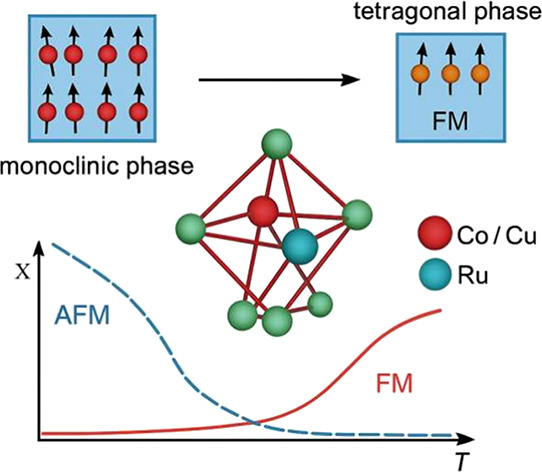

## 1 Introduction

Double perovskite oxides with the general formula A_2_BB′O_6_, where A is a rare-earth or alkaline-earth element and B/B′ are transition metal ions, have attracted significant attention due to their highly tunable crystal structures and rich variety of magnetic behaviors.^1–4^ These properties are strongly dependent on the degree of B-site cation ordering, which can range from fully ordered (rock-salt or layered) to completely disordered arrangements.^5,6^ The extent of this ordering is influenced by factors such as the difference in ionic radii and valence states of the B-site cations, as well as synthesis conditions like temperature and atmosphere.^7,8^ Magnetism in these systems varies widely, from ferromagnetic (FM) to spin-glass and antiferromagnetic (AFM) ground states, driven by the interplay of superexchange interactions, structural distortions, and multivalent cation configurations. Compounds such as La_2_MnRuO_6_ (FM), La_2_FeRuO_6_ (spin-glass), and La_2_CoRuO_6_ (AFM) illustrate the sensitivity of magnetic ground states to B-site composition.^9–11^

Theoretical understanding of such systems remains challenging due to their complex electronic and magnetic interactions. Recent studies on La_2_CuRuO_6_, for instance, highlight the difficulty in clearly identifying magnetic ground states despite well-characterized structural features.^3,12,13^ Building on this, recent work by Haque et al.^14^ explored Cu substitution in La_2_MnCoO_6_, revealing that Cu doping not only induces a structural transition, from monoclinic to rhombohedral, but also significantly alters the valence states and magnetic interactions of Mn and Co. The replacement of smaller Mn^4+^ ions with larger Jahn–Teller-active Cu^2+^ (and the accompanying mixed valence states such as Co^2+^/Co^3+^ and Mn^3+^/Mn^4+^) leads to complex superexchange pathways and a shift from long-range FM to competing AFM interactions.

At higher Cu concentrations (*x* ≥ 0.2), AFM ordering dominates and helps suppress the antistitute-disorder-induced magnetic frustration present in the undoped system. This shift is attributed to the structural distortions and multivalent cation effects, offering insights into how targeted B-site doping can be used to tune magnetic ground states in double perovskites. These findings emphasize the importance of Jahn–Teller distortions, charge compensation mechanisms, and cation size mismatches in controlling structural and magnetic phase behavior.^7,15^ Drawing from these insights, this work investigates Cu-doped La_2_CoRuO_6_ (LCRO) to understand how Cu^2+^ substitution at the B-site modulates its magnetic structure, valence states, and the potential suppression of frustration-induced effects observed in undoped LCRO. In its pristine state, LCRO exhibits a rock-salt type B-site ordering between Co^2+^ and Ru^4+^ ions, crystallizing in the monoclinic *P*2_1_/*c* (no. 14) space group.^16^

The extent of the B-site magnetic ordering is typically inferred from the presence or absence of characteristic Bragg peaks at low diffraction angles. Morimura and Yamada^16^ recently revealed that a high degree of B-site ordering gives rise to additional Bragg reflections near 2θ ≈ 5.25 and 5.3°. This pronounced ordering is correlated with an AFM transition, observed at a Néel temperature (*T*_N_) of approximately 25 K.^17^ The introduction of Cu into the LCRO influences both structural ordering and magnetic interactions. Here, the focus is on the compositions LC_(1–*x*)_RO:Cu_*x*_ (*x* = 0, 0.05, and 0.3), where a notable structural transition from monoclinic to tetragonal symmetry emerges as early as 5% Cu doping, highlighting the sensitivity of the perovskite framework to B-site perturbations. Beyond structural and magnetic characterization, we also explore the electronic properties of both the pristine and Cu-doped LCRO through first-principles computational methods.

These electronic structure calculations provide critical insight into the superexchange mechanisms that govern the evolution of magnetic behavior across doping levels. The coupling among structural distortions, cation valency, and electronic configuration forms the basis for understanding the magnetic phase evolution in this system. This integrated experimental–computational approach offers a comprehensive understanding of how targeted Cu doping modulates the structure–property relationships in LCRO-based double perovskites.

## 2 Experimental Section

### 2.1 Solid-State Synthesis

Pristine LCRO (*x* = 0) and Cu (*x* = 0.05 and 0.3) doped polycrystalline samples were synthesized by a conventional high-temperature solid-state reaction method under controlled conditions of temperature and time. For the pristine sample, stoichiometric proportions of high-purity (99.99%) powders of lanthanum­(III) oxide (La_2_O_3_), cobalt oxide (Co_3_O_4_), and ruthenium­(IV) oxide (RuO_2_) were mixed.

To dope the sample, *x* = 0.05 and 0.3 portions of copper­(II) oxide (CuO) powder were used to substitute equal portions of Co_3_O_4_ in LC_(1–*x*)_RO:Cu_*x*_.

The separate samples were mixed thoroughly with a pestle and mortar and preheated at 1100 °C for 24 h in an air atmosphere. The preheated powders were sintered again at 1200 °C for 50 h with intermediate grinding before any characterization processes could be employed (Figure 1).

**Figure 1:**
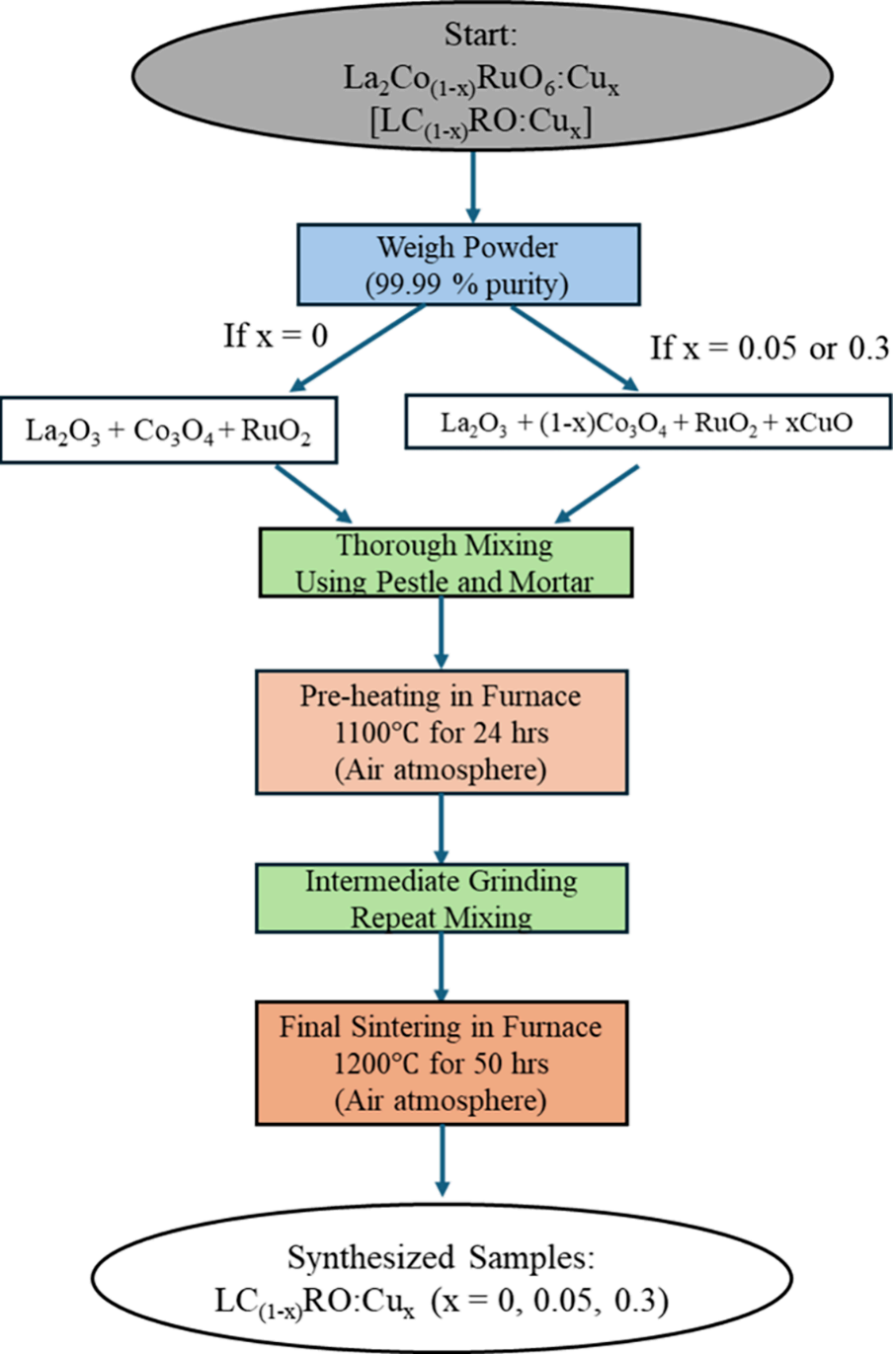
Flowchart diagram illustrating synthesis of LC_(1–*x*)_RO:Cu_*x*_ (*x* = 0, 0.05, and 0.3) powder.

### 2.2 Characterization

Phase purity and crystal structure of the synthesized samples were examined via powder X-ray diffraction (XRD) using a diffractometer equipped with Cu Kα radiation (λ = 1.5406 Å). Structural analysis and determination of lattice parameters were carried out through Rietveld refinement by using the EXPO2014 software. The finalized crystal structures were rendered with VESTA for three-dimensional visualization. Elemental composition and homogeneity were assessed through energy-dispersive spectroscopy (EDS). Alternating current heat capacity (AC-Cp) measurements were performed by using an AC calorimetry setup. For these measurements, the samples were prepared by homogeneously mixing equal masses of the powder and N-grease into a paste, which was then applied to the AC platform’s measurement membrane. The power and frequency parameters were optimized by analyzing the system’s response at ambient temperature. Magnetic properties were probed using a cryogenic physical property measurement system (PPMS) equipped with a vibrating sample magnetometer (VSM) module.

### 2.3 Computational

The partial density of states (PDOS) and total density of states (TDOS) of monoclinic LCRO (space group *P*2_1_/*c*) and a Cu-doped LCRO (*x* = 0.05 and 0.3) tetragonal (*I*4*m*) were respectively computed using density functional theory (DFT) within the CASTEP code.^18,19^ Employing the generalized gradient approximation (GGA) in the Perdew–Burke–Ernzerhof (PBE) formulation for the exchange-correlation functional and a collinear spin-polarized DFT + U framework to capture electron correlations in Co and Ru 3*d*/4d orbitals via the nonmagnetic O 2p.^20,21^ Electronic correlation effects were accounted for using the GGA + U method in its rotationally invariant form, with effective Hubbard parameters of *U*_eff_ = 3.0 eV for Co and Cu and 2.5 eV for Ru. On-the-fly generated (OTFG) ultrasoft pseudopotentials with scalar relativistic Koelling–Hamann treatment were applied, and a Γ-point-only (2 × 2 × 2) k-point mesh was used, given the large unit cell.^22^ Structural relaxation was performed prior to electronic calculations with a 489.80 eV plane-wave cutoff, energy convergence of 1 × 10^–6^ eV/atom, and force tolerance of 0.01 eV/Å. Orbital-projected PDOS and band structures were derived from the relaxed geometry to analyze contributions from La, Co, Ru, and O atoms. Smearing techniques ensured smooth PDOS profiles.

## 3 Results

### 3.1 Structural Analysis

Room temperature XRD patterns in Figure 2a–c were used to study the crystal structure and phase purity of a pristine LCRO and LC_1–*x*_RO:Cu_*x*_ (*x* = 0, 0.05, and 0.3) compounds. The highly crystalline patterns with their respective Rietveld refinements were fitted by using different crystal structures. The pristine sample was refined with a monoclinic structure of the *P*2_1_/*c* space group no. 14 in Figure 2a.^16^ The inset of Figure 2a shows the ordering of Ru and Co octahedra in their distinguishable 2*a* and 2*d* sites, respectively. Substitution of Co with Cu (*x* ≥ 0.05) results in a transition of crystal structure to form tetragonal *I*4/*m* of space group no. 87 in Figure 2b,c.^23^ This substitution occurs at the 2*a* site that is shared by Co^2+^/Cu^2+^ octahedra, while RuO_6_ is at the 2*b* site of the structure.

**Figure 2:**
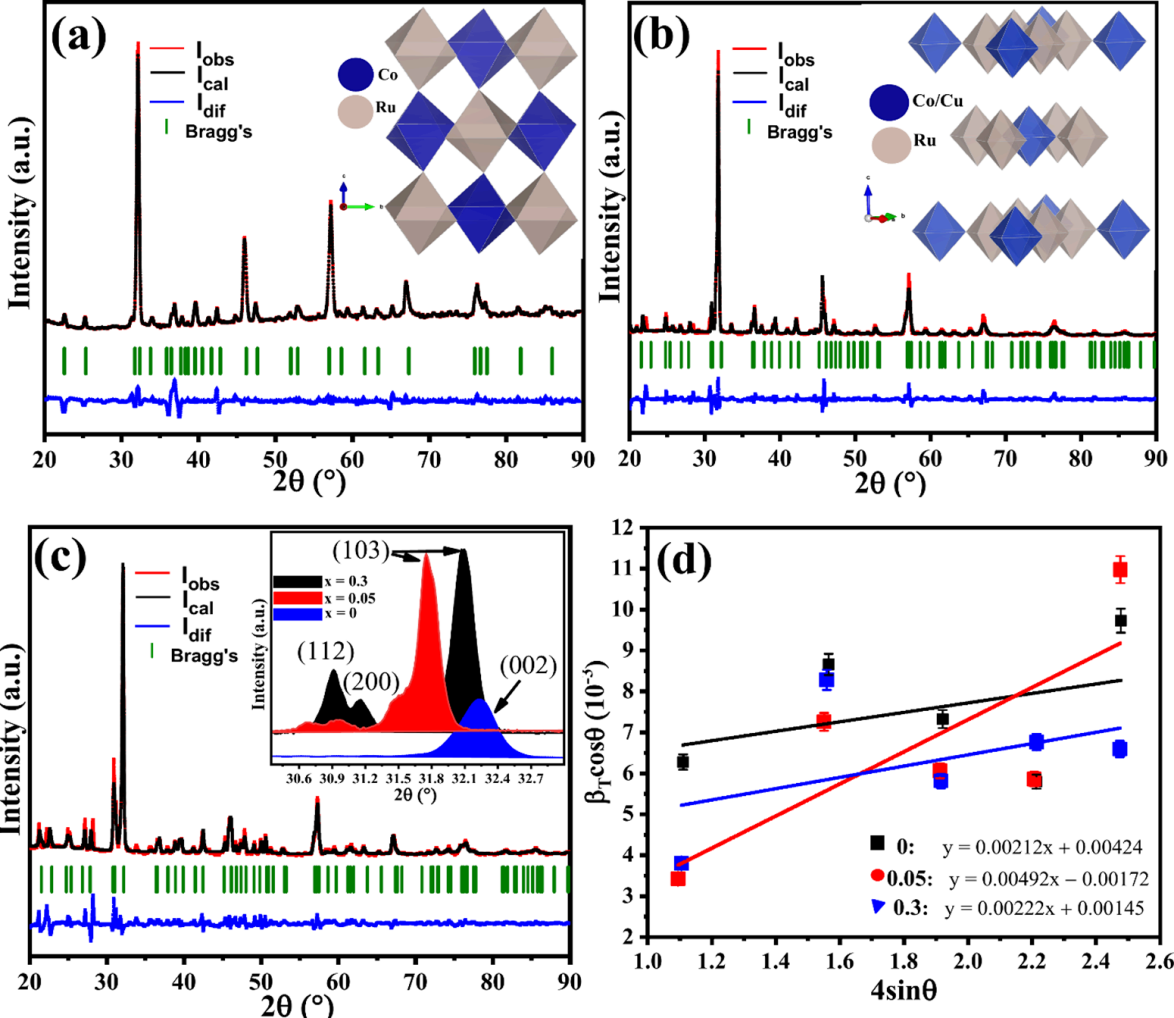
Powder XRD spectrum (red solid line) with the Rietveld refinement fit (black line). The difference curve is shown by a blue solid at the bottom of the patterns, while the green ticks are Bragg peaks of the different compound of the (a) host LCRO (*x* = 0) pattern fitted with a monoclinic *P*2_1_ *c* structure (inset). Doped LCRO with (b) *x* = 0.05 fitted with a tetragonal *I*4*/m* crystal structure shown in the inset. (c) *x* = 0.30 of Cu including an inset showing the XRD patterns of all the samples. (d) Williamson–Hall effect of the different samples with tensile strain shown as the linear plot slope.

The all-atom unit cells of the pristine monoclinic structure and doped tetragonal structure are also included in the Figure S1. The reliability factors from Rietveld refinement in Table 1 confirm good consistency between the refined and experimental XRD data for all samples. The initial observation upon doping at 0.05 is a decrease in *R*_p_/*R*_wp_ values relative to 0, suggesting improved order and grain growth. At 0.3, these values are increased with structural complexity due to higher Cu content. This is likely due to unmodeled effects such as octahedra distortions.

**Table 1:** Unit Cell and Positional Parameters after the Rietveld Refinement of the Crystal Structure from XRD Data at RT of *x* = 0, 0.05, and 0.30 Samples with an Error of ± 0.01

sample space group	*x* = 0*P*2_1_ *c* (#)	*x* = 0.05*I*4/*m* (#)	*x* = 0.30*I*4/*m* (#)
Cell Parameters			
*a* (Å)	5.49		
		8.67	8.72
*b* (Å)	5.62		
*c* (Å)	9.49	12.73	12.75
α (°)	89.91		
β (°)	125.06	90	90
γ (°)	90.41		
V (Å^3^)	245.19	955.86	968.24
Fitting Parameters (Rietveld)			
*R*_p_	2.972	2.035	2.417
*R*_wp_	4.052	3.259	3.712
χ^2^	4.324	4.764	4.588
Strain and Crystallite Size (W–H)			
ε (10^–3^)	2.12	4.92	2.22
*D* (nm)	32.70	80.61	95.62

The observed consistency in Rietveld fitting across all phases confirms the stability of the structural transformation observed in this work. The insert of Figure 2c shows comparative XRD spectra of the three samples in a region between 30.4 and 32.9°. The shift of the peak from 32.2 (0) to 31.8° (0.05) reflects lattice expansion due to the substitution, while a return to 32.1 at 0.3° suggests partial relaxation and ordering in the emerging tetragonal phase. Transition from lower symmetry (monoclinic) phase to higher symmetry (tetragonal) phase causes peak split (previously forbidden reflections are allowed in the tetragonal phase).^24,25^ The new reflections [(112)/(200) peaks] intensify with increasing Cu content due to the enhanced ordering that comes with grain growth.

The lattice strains and crystallite sizes of the samples were obtained by using the Williamson–Hall (WH) fit.

$$\beta_{T}\cos\;\theta =4\varepsilon \sin\theta +K\lambda /D$$ 1

where β_*T*_ is the average full-width at half-maximum (fwhm) of the intense Bragg peaks, *K* = 0.9 is a shape factor constant, λ *=* 0.154 nm is the source of the X-ray wavelength, *D* represents the crystallite size, and the term ε in eq 1 is the magnitude of strain.^26^ A WH plot is obtained from β_T_ cos θ against 4εsinθ for each sample with their respective error bars, as illustrated in Figure 2d, with the *y*-intercept representing the crystallite size.

The slope of the linear fit of the plots represents the strain. The calculated values of *D* (nm) and ε are shown in Table 1 for the respective samples. Cell parameter results in Table 1 reveal a significant increase in volume of material with the introduction of Cu^2+^ in the pristine sample. A nearly 4-fold volume expansion due to the structural transition is further expanded for 0.3 as a consequence of crystallographic expansion and structural distortions.

The strain is also enhanced by a ratio of approximately 2.4, as the structure transitions from monoclinic to tetragonal. This suggests the transition occurs to accommodate the resulting strain, while reducing the lattice energy that occurs due to mismatch brought about by doping.^27,28^ While 0.3 increases the volume, the structure is more relaxed and stable in the tetragonal phase, reducing the strain and distortion. The increase of the crystallite size during the transition by approximately the same ratio (∼2.5 ratio) as the strain is further evidence of a strain-driven (tensile) phase transformation that encourages grain coarsening, with the tetragonal phase being energetically favorable at 0.05 doping.^29^

### 3.2 Morphological Analysis

The morphology, size distribution, and elemental composition of the compounds were examined using SEM and EDS, respectively. The pristine sample micrograph in Figure 3a shows well-rounded particles that appear to sinter. The effect of 0.05 in Figure 3c is an increased particle size with less adhesion of the spherical particles. At this concentration, uniformity of the particle is improved with less sintering due to reduced disorder and strain at the surface, thereby lowering the surface energy. Increasing the concentration even further (0.3) in Figure 3e results in larger particles as discussed in Section 3.1; however, this enlargement comes with irregularity in the morphology of the particles. These flaky-like particles that appear to be brittle might suggest anisotropic stress that increases surface energy due to the preferential orientation of the (112) plane in the inset of Figure 2c.^30^ The corresponding size distribution histograms show the average particle diameter (<*D*_*x*=_>) of the compounds in Figure 3b,d,f at pristine, 0.05, and 0.3 to be 3.2 ± 0.6, 3.3 ± 0.8, and 8.8 ± 1.9 μm, respectively. While the particle size increases with doping concentration in comparison to the crystallite size, the 0.05 results indicate intragranular crystallite growth, outpacing the increase in overall particle size. However, this process does not markedly increase the overall particle size. Instead, the particle exhibits enhanced crystallinity and reduced sintering as a result of strain-relieving mechanisms at the surface. At 0.3, the particle size is substantially increased due to the large Cu content that allows full stabilization in the tetragonal phase. The observed results confirm the strain-driven crystallite growth mechanism and a structural phase transition that governs the particle morphology. Insets of Figure 3a,c,e show variation in Cu^2+^ content (yellow).

**Figure 3:**
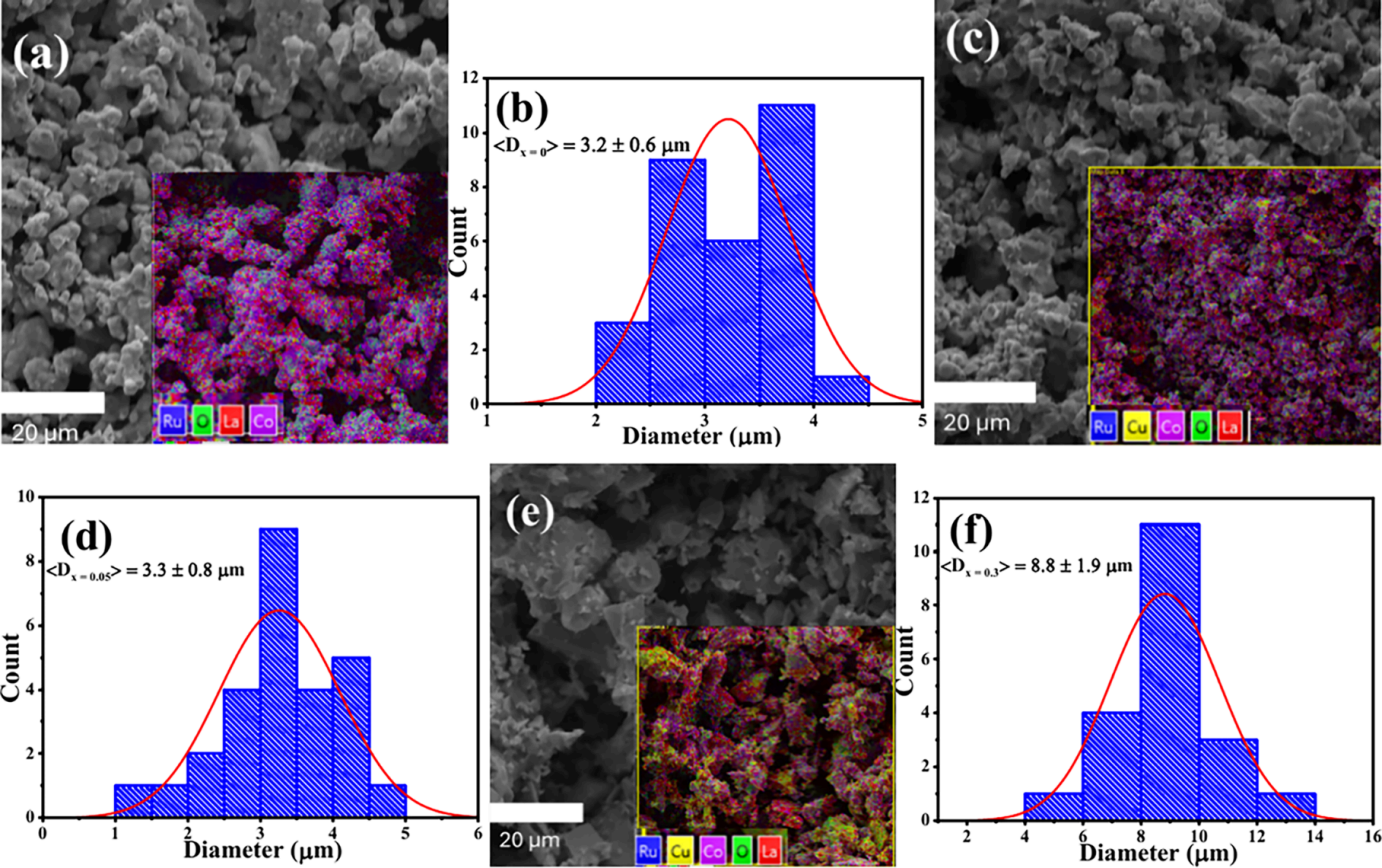
Surface morphology SEM micrographs with elemental mapping (insets) of the (a) pristine (*x* = 0), (c) *x* = 0.05, and (e) *x* = 0.3 of the La_2_Co_(1–*x*)_RuO_6_:Cu_*x*_ compounds, with their respective size distribution histograms: (b) *x* = 0, (d) *x* = 0.05, and (f) *x* = 0.3.

Cu^2+^ is successfully incorporated into the lattice in 0.05 and 0.3 as shown in the surface elemental and weight percentage composition results in Figure 4a,b, respectively. The increase in Cu content from 1.9 to 6.8% confirms the intentional substitution of Co as it decreases from 30.2 to 9.8%, aligning with the 2a site substitution of Co^2+^.

**Figure 4:**
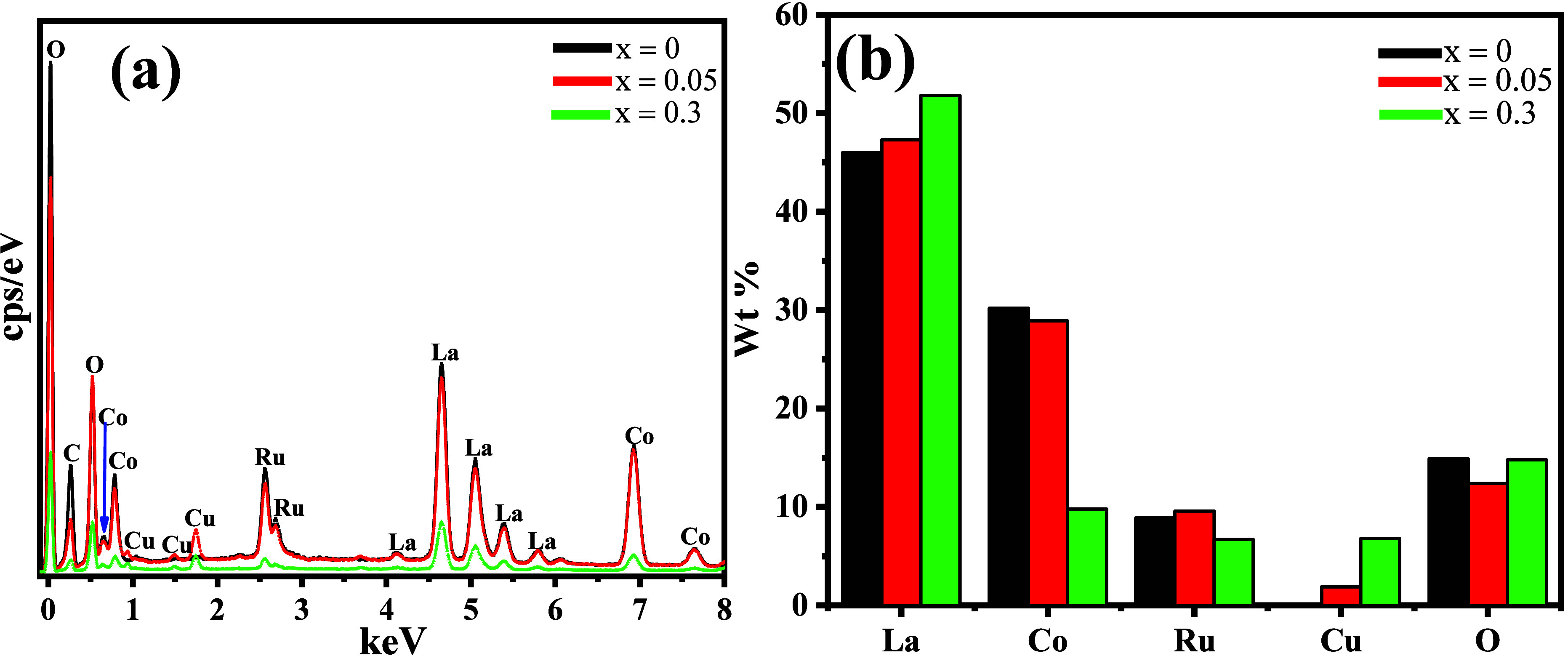
EDS spectra showing the (a) surface elemental composition of the La_2_Co_(1–*x*)_RuO_6_:Cu_*x*_ compounds and (b) their varying weight percentage content.

The slight variation in the Ru content across the different concentrations is possibly due to mixed valence of Ru or slight occupancy shifts.^31,32^ La content also increases with Cu, suggesting possible surface exposure or alteration of the surface chemistry due to disruption of the Co site with Cu enrichment.^33^ Disruption of the metal–oxygen octahedra with structural change and strain is likely the cause of the variation in O content with enhanced strain reducing O.^34,35^ There are thus no observed impurities in this work, with the occurrence of the expected ions of La, Co, Ru, and Cu.

### 3.3 Compositional Analysis

The surface chemical composition and electronic structure of LC_(1–*x*)_RO:Cu_*x*_ were systematically examined using XPS in Figure 5a–h. The C 1s-calibrated (average ≈ 285.0 eV) spectra displayed in this work are those of elements such as the Co 2p, Cu 2p, and O 1s. The XPS surface chemical quantification data with chemical IDs for the respective samples are shown in Section S2 of the Supporting Information.

**Figure 5:**
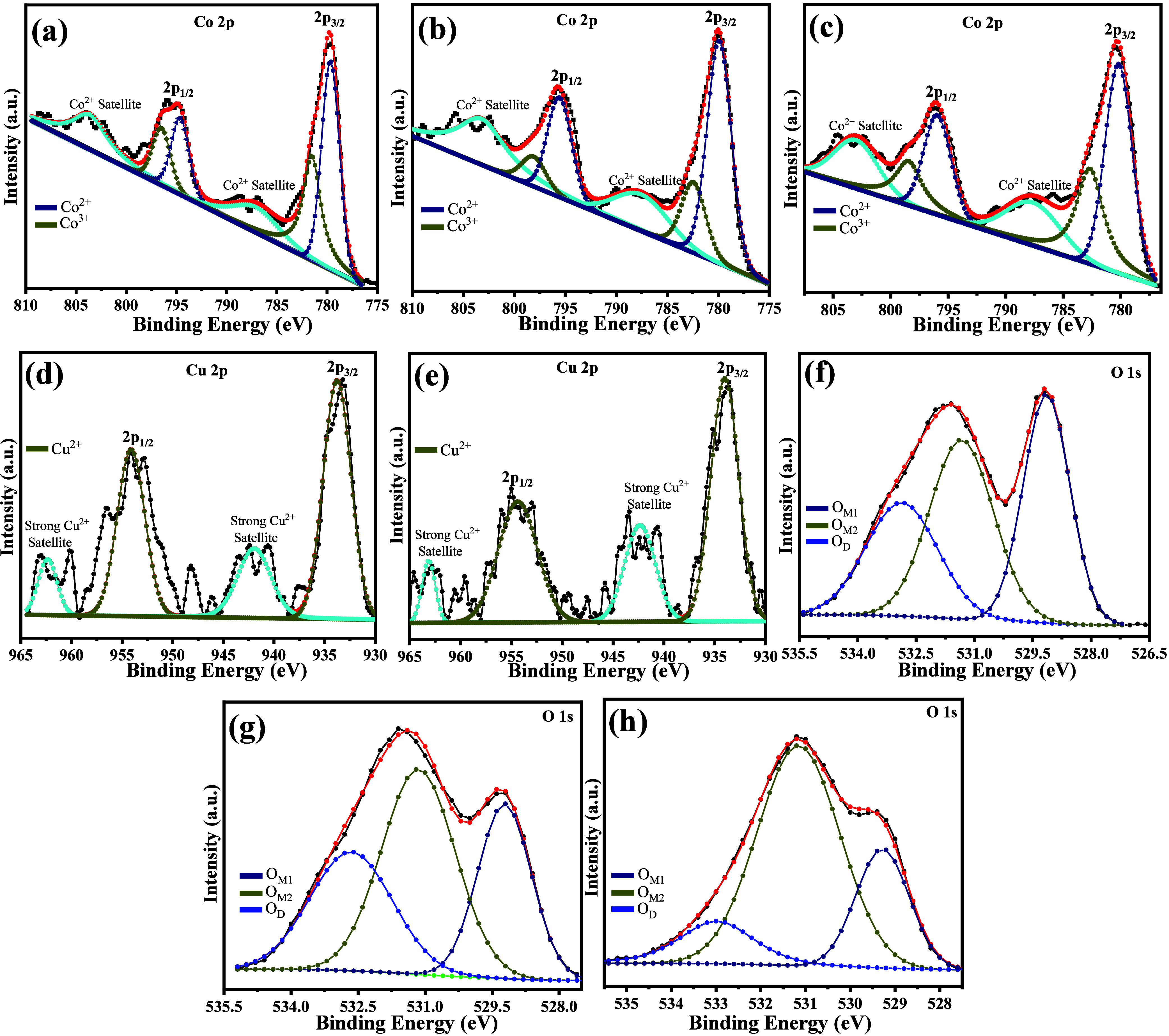
X-ray photoelectron spectroscopy (XPS) core-level spectra of La_2_Co_(1–*x*)_RuO_6_:Cu_*x*_ compounds showing (a–c) Co 2p, (d–e) Cu 2p, and (f–h) O 1s regions for pristine, *x* = 0.05, and *x* = 0.3 samples, respectively. Progressive Co 2p and Cu 2p binding energy shifts with Cu incorporation indicate charge transfer and enhanced Co–O–Ru hybridization. Deconvolution reveals mixed Co^2+^/Co^3+^ and Cu^2+^ states, while the O 1s spectra show reduced defect-related components at higher Cu content, evidencing improved crystallinity and oxygen lattice uniformity.

The Co 2p core-level spectra in Figure 5a–c reveal two primary components alongside two minor satellite features. The primary Co doublets are those of cobalt oxides corresponding to 2p_3/2_ and 2p_1/2_ at binding energies (BEs) of 780.2 and 795.9 eV, respectively. The spin–orbit doublet of 2p_3/2_ and 2p_1/2_ is deconvoluted into Co^2+^ and Co^3+^ peaks with their positions summarized in Table 2. There is an observed peak shift here with broadening of the spin–orbit splitting of the doublet from 15.0 eV in the pristine sample to 15.7 and 15.8 eV for the 0.05 and 0.3 samples, respectively. The progressive broadening observed with Cu incorporation indicates pronounced alterations in the local electronic structure of Co, driven by crystal field perturbations and charge redistribution among Co, Cu, and Ru cations.^36^ Replacing high-spin Co^2+^ (3d^7^) with Jahn–Teller-active Cu^2+^ (3d^7^) enhances metal–oxygen covalency and induces lattice distortions that modify crystal field splitting and Co–O–Ru hybridization. These changes cause a Co 2p binding energy shift, evidencing a partial charge transfer from Co^2+^ to Cu^2^^+^ and Ru^4+^.

**Table 2:** Summary of Peak Positions From the Deconvoluted Spectra of the La_2_Co_(1–*x*)_RuO_6_:Cu_*x*_ compounds shown in Figure 5 with an Error of ± 0.01

	Co 2p_3/2_ (eV)		Co 2p_1/2_ (eV)		Cu^2+^ 2p (eV)		O 1s (eV)		
sample	Co^2+^	Co^3+^	Co^2+^	Co^3+^	Cu 2p_3/2_	Cu 2p_1/2_	O_M1_	O_M2_	O_D_
0	779.7	781.5	794.6	796.5			529.1	531.3	532.9
0.05	779.9	782.4	795.6	798.1	933.8	954.1	529.2	531.1	532.7
0.3	780.2	784.7	795.9	798.4	934.0	954.4	529.3	531.2	533.0

The presence and persistence of two intense satellite peaks throughout the samples, in the vicinity of the spin orbit doublet, is evidence of the dominance of the Co^2+^ of cobalt oxides.^37,38^ While Co^2^^+^ ions in spinel structures often occupies tetrahedral sites with higher BEs due to local crystal field and final state effects, in our double perovskite oxides structures Co ions are octahedrally coordinated with Co^3^^+^ consistently showing higher Co 2p BEs than Co^2^^+^.^37,39^ Figure 5d,e shows the deconvoluted Cu 2p_3/2_ and 2p_1/2_ doublet doped samples with their corresponding BEs summarized in Table 2 Cu in both the samples further has intense satellite peaks at 941.9 and 962.4 eV in 0.05, and 942.3 and 963.1 eV in 0.3.

The presence of these satellite peaks is further evidence of the Cu^2+^ state.^40,41^ There are observed peak shifts with increasing Cu substitution to higher BE in both the Co 2p and Cu 2p peaks, which further support the results discussed in the Structural Analysis section. This shift is attributed to a number of structural factors, such as enhanced crystal field effects, reduced electron shielding due to strain-modified electronic modification, improved crystallinity, and octahedral coordination environment. Deconvoluted O 1s XPS spectra presented in Figure 5f–h are fitted with three peaks. The first two low BE correspond to the metal oxygen bonds (O_M_), and a higher BE indicates the presence of defect (O_D_) sites with their relative positions shown in Table 2.^42,43^

Due to mixed valence states and a structurally inhomogeneous oxygen environment, there is a clear split of the two metal oxygen bonds that progressively diminishes with increasing Cu^2+^ concentration. The annihilation of the split at 0.3 is accompanied by a significant reduction of the defect-associated peaks is a consequence of the improved crystallinity, reduced surface disorder, and a more uniform Co/Cu–O–Ru network.^44^

### 3.4 Superexchange Mechanisms

To understand the magnetic interactions in LCRO and Cu-doped LCRO, we computed spin-polarized partial densities of states (PDOS) for Co 3d, Ru 4d, and O 2p orbitals, considering the various oxidation states identified in Section 3.3 from XPS analysis (Figures 6a–c). Prior to these calculations, geometry optimization was carried out for all neutral formula units to relax the structures and ensure accurate electronic configurations. The compositions considered were La_2_^3+^Co^2+^Ru^4+^O_6_^2–^ and La_2_^2+^Co^3+^Ru^5+^O_6_^2–^, representing the different possible oxidation states in the samples.

**Figure 6:**
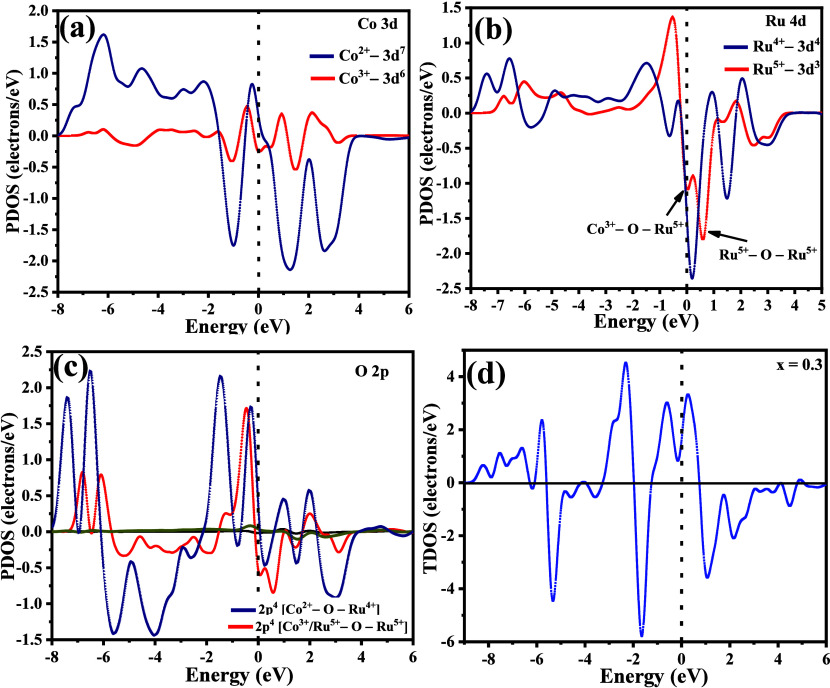
Spin-polarized partial and total densities of states (PDOS and TDOS) illustrating magnetic exchange mechanisms in LCRO and Cu-Doped LCRO. (a) Co 3d PDOS, (b) Ru 4d PDOS, and (c) O 2p PDOS highlighting spin-resolved asymmetry consistent with superexchange mediation between Co/Ru cations, emphasizing enhanced covalency in higher oxidation states, and (d) spin-resolved TDOS for 0.3 Cu-doped LCRO revealing enhanced up-spin polarization and coexisting FM and AFM contributions due to Cu^2^^+^ substitution.

For La_2_^3+^Co^2+^Ru^4+^O_6_^2–^, high-spin Co^2+^ (S = 3/2) and low-spin Ru^4+^ (S = 1) show strong spin asymmetry near the Fermi level (E_F_), primarily in the down-spin channel. This is a clear signature of robust AFM superexchange through Co^2+^–O–Ru^4+^ pathways. In contrast, Co^3+^ (S = 0) exhibits symmetric spin states, confirming its nonmagnetic character and negligible contribution to magnetic interactions. While Co^3+^ and Ru^5+^ (S = 3/2) coexist in La_2_^2+^Co^3+^Ru^5+^O_6_^2–^, the absence of a magnetic moment on Co^3+^ prevents significant Co^3+^–O–Ru^5+^ coupling. The Ru 4d PDOS (Figure 6b) shows strong spin asymmetry, with a deeper peak around −1.8 e^–^/eV attributed to AFM Ru^5+^–O–Ru^5+^ interactions and a shallower feature near −1.09 e^–^/eV likely corresponding to weakly interacting Co^3+^–O–Ru^5+^ pathways. The pronounced up-spin peak (∼1.4 e^–^/eV) further emphasizes the spin imbalance characteristic of AFM Ru^5+^ configurations.

The bridging 2p O orbitals (Figure 6c) exhibit spin-resolved asymmetry consistent with superexchange activity, where Co^3+^/Ru^5+^–O–Ru^5+^ pathways show slightly increased down-spin intensity near *E*_F_. This indicates enhanced covalency and orbital overlap in higher oxidation states, though the contribution to overall magnetism is weaker than that of Co^2+^–O–Ru^4+^.^45,46^ The total density of states (TDOS) for 0.3 Cu-doped LCRO in its most stable FM Cu^2+^ (3d^9^, S = 1/2) configuration is shown in Figure 6d.^47^ Here, a pronounced spin asymmetry appears, with enhanced up-spin intensity near *E*_F_. The conduction band shows higher up-spin TDOS compared to the valence band, while the down-spin states are shifted further from *E*_F_, reflecting local magnetic inhomogeneities.

This asymmetry suggests that FM interactions introduced by Cu^2+^ coexist with the intrinsic AFM framework.^48,49^ Substituting Co^2+^ with Cu^2+^ subtly perturbs the original Co^2+^–O–Ru^4+^ AFM pathways. Because Cu^2^^+^ has a lower spin, it may favor FM interactions with Ru^4^^+^ or neighboring Cu^2^^+^ ions, especially in distorted octahedral environments, giving rise to competing Cu^2+^–O–Ru^4+^ or Cu^2+^–O–Cu^2+^ exchange paths.^50^ However, the TDOS indicates that these FM contributions, while present, do not overcome the dominant AFM interactions.

### 3.5 Magnetic Analysis

Temperature-dependent zero-field cooling-warming (ZFCW) magnetic susceptibility, χ­(*T*), curves obtained at a field of 0.5T for the different compounds are shown in Figure 7a–c in the 0–300 K temperature range. At low temperatures, there is an observed AFM transition, T_N_, for the LCRO sample is at 28.7 K. Often attributed to dominance of long-range AFM order between Co and Ru, the transition is widely reported to be around 25 K for similar structures.^8,16,51^ The presence of mixed oxidation states discussed in Section 3.3 suggests multiple magnetic exchange pathways, deviating from the canonical Co^2^^+^–O–Ru^4^^+^ AFM superexchange that dominates in the ideal double perovskite structure with *P*2_1_/*c* symmetry. Our first-principles calculations in Section 3.4 also reveal that Ru^5+^–O-Ru^5+^ AFM interactions may emerge locally due to charge imbalance or clustering of Ru^5+^, further complicating the magnetic landscape. This complex magnetic topology is further influenced by cationic disorder at the B-site, wherein the random distribution of Co and Ru ions alters the local bonding geometry and electronic bandwidth.

**Figure 7:**
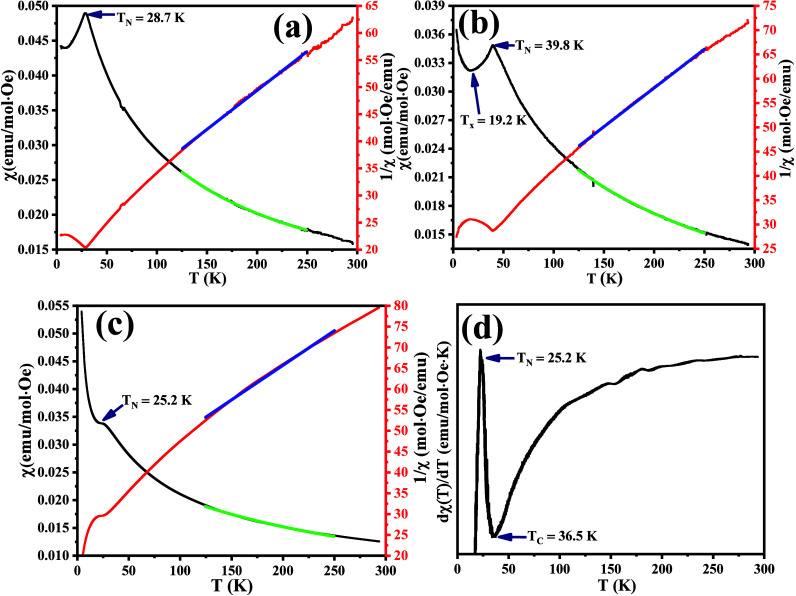
Temperature-dependent zero-field cooling–warming (ZFCW) magnetic susceptibility, χ­(*T*), curves obtained at a field of 0.5T of the (a) pristine (*x* = 0), (b) *x* = 0.05, and (c) *x* = 0.3 samples, with their respective inverse susceptibility, χ­(*T*)^−1^, curves on the right (red). All the curves are fitted with two models (125 ≤ *T* ≤ 250) for estimating the effective magnetic moment, with the χ­(*T*) vs T curves fitted with a green fit of a modified Curie–Weiss (CW) mode and the χ­(*T*)^−1^ vs *T* curves fitted with a blue fit of the normal CW model. (d) First-order derivative of the 0.3 sample with the resultant magnetic transitions.

Structural disorder tends to enhance the number of Co^2^^+^–O–Ru^4^^+^ interactions, either via partial charge redistribution or by promoting local distortions that favor AFM alignment.^17,52,53^ This results in an increase in the effective AFM coupling strength, raising the *T*_N_ temperature. This is consistent with prior reports suggesting that B-site disorder can enhance magnetic interactions in double perovskites by tuning the orbital overlap and superexchange energy scales.^16^ Low-concentration substitution of Cu (0.05) in the pristine sample results in a significant shift of the *T*_N_ to 39.8 K, and at higher concentrations (0.3), it goes down to 25.2 K. The enhancement at 0.05 can be explained by both structural phase transition and strain-enhanced effects. The initial substitution of Cu at the Co site causes lattice expansion and internal stress, distorting the local octahedra, strengthening anisotropic interactions, and reducing magnetic frustration. For 0.3, *T*_N_ drops back to 25.2 K, which is even lower than that of the pristine sample.

In Figure 7b, we observe a secondary anomaly at 19.2 K (*T*_*x*_), which is probably due to a local spin reorientation or partial magnetic cluster freezing caused by lattice strain and magnetic competition between Co^2^^+^–O–Ru^4^^+^ (AFM) and emergent Cu^2^^+^–O–Ru^4^^+^ (FM-like) interactions. The substitutional strain and mild disorder at low Cu levels introduce local anisotropy and inhomogeneity, sufficient to trigger such a transition.^54^ Relaxation of the lattice due to reduced stress, along with increased site disorder introduced by Cu atoms, enhances competing exchange paths between Co^2+^–O–Ru^4+^ and Cu^2+^–O–Ru^4+^, reducing the long-range ordering in 0.3. This dilution with competing exchange pathways dilutes the magnetic moment carriers and reduces *T*_N_. The strong emergence of Cu^2^^+^–O–Ru^4^^+^ or Cu^2^^+^–O–Cu^2^^+^ as alternative pathways observed in Section 3.4 introduces local magnetic inhomogeneities in 0.3, with a FM Curie transition (*T*_C_) at temperatures above *T*_N_ in Figure 7d.

This figure depicts a first-order derivative of χ­(T), [dχ­(*T*)/d*T*], with *T*_C_ being the temperature where spin coupling forces of the FM moments are destroyed with increasing temperature. Warming up to high temperatures beyond 125 K leads to complete dominance of paramagnetic (PM) spins in all of the compounds. The linear behavior observed in the inverse susceptibility curves, χ­(*T*)^−1^, shown in Figure 7a–c as a blue line (125 ≤ *T* ≤ 250), is consistent with the Curie–Weiss (CW) law, given by χ­(*T*)^−1^ = (*T* – θ_CW_)/*C*. Here, *C* represents the Curie constant, which is directly linked to the number of unpaired electrons. This relationship provides a means to extract the effective magnetic moment (μ_eff_) per ion, expressed in units of Bohr magneton μ_B_. The resulting μ_eff_ values for the three samples are summarized in Table 3 below.

**Table 3:** Comparison of the CW and a Modified CW Fits to Estimate the Effective Magnetic Moment of the Pristine, , 0.05, and 0.3 Cu-Doped Samples^a^

	CW fit			modified CW fit				
sample	θ_CW_ (K)	C	μ_eff_ (μ_B_)	R (nm)	η (10^–4^)	θ_v_(K)	C_v_	μ_eff_ (μ_B_)
0	–91.1	5.6	6.7	1600	5.6	–64.0	3.9	5.6
0.05	–99.8	4.8	6.2	1650	5.5	–98.5	4.8	6.2
0.3	–97.8	4.2	5.8	4400	2.1	–98.6	4.2	5.8

a The modified version of the classical law considers size and shape effects with an error of ± 0.01.

Our results show an overestimated effective moment value for the pristine sample when compared to the theoretical values, with a reduction of the effective moment as we increase the Cu concentration.^55,56^ Morimura and Yamada^57^ have previously attributed the overestimation in the experimental magnetic moment to unquenched orbital moments in Co^2+^ ions. While the CW law has long served as a foundational model in classical magnetism, predicated on the assumptions of a bulk, isotropic, and homogeneous material with uniformly interacting magnetic moments described by a classical mean-field approach.^58–63^ However, this framework overlooks critical factors such as quantum mechanical effects, surface contributions, shape-induced anisotropy, and finite-size constraints.^64–66^ These limitations render it inadequate for accurately describing magnetic behavior at the nanoscale, particularly in systems like quantum dots, where quantum and surface phenomena play a dominant role. In our recent publication on quantum size effects on magnetism, we explored the introduction of a modified CW law (eq 2) that considers finite-size phenomena along with shape,^66^ defined with

$$\chi (T)=\eta \frac{C_{\mathrm{surf}}}{T-\theta_{\mathrm{surf}}}+(1-\eta )\frac{C_{\mathrm{vol}}}{T-\theta_{\mathrm{vol}}}$$ 2

where η is a surface fraction term that is dependent on both the size and shape of the material. For a spherical particle with $V=\frac{4\pi }{3}r^{3}$ , the fraction becomes $\eta =\frac{3t}{R}$ with *t* representing the thickness of the surface atomic layer (∼0.3 nm) and *R* the radius of the sphere.^1,67,68^ A fit of eq 1 in Figure 5 with the light-green fit (125 ≤ *T* ≤ 250), assuming that as *R* → ∞, η → 0 and (1 – η) → 1, leading to the normal CW equation. The resulting magnetic moment values obtained from this version are comparable to a slight difference in the pristine sample. For this sample, this model results in a lower effective moment, which better matches the theoretical prediction from bulk LCRO moments.

Improved magnetic ordering (due to larger crystallites and strain) and the introduction of a Cu–O–Ru FM coupling enhance the Cu^2+^ (d^9^) contribution, causing an increase in μ_eff_. At large particles with relaxed strain, the structural and magnetic environments are sufficiently homogeneous, completely eliminating the surface effects with increasing particle radius. The Weiss constant θ_CW_/θ_v_ from the two models shows a similar trend but differs in value, with the normal CW law showing higher values (assumes homogeneity). Once again, these results show that for a smaller particle with a smaller η, the CW model overestimates the Weiss constant skewed by surface disorder, strain, and inhomogeneity. Meanwhile, θ_v_ reveals the true intrinsic interaction strength of the core atoms. This suggests that Weiss constants in the two magnetic models are sensitive to structural inhomogeneity (accounting for surface effects).

## 4 Conclusions

In this investigation, we have conducted a thorough examination of the substitution of Cu^2^^+^ for Co^2^^+^ in the double perovskite LCRO characterized by a monoclinic *P*2_1_/*c* structure. The incorporation of 0.05 Cu^2^^+^ has led to a structural transformation resulting in the formation of a tetragonal *I*4/*m* phase accompanied by a reduction in strain. The phenomenon of crystal growth being disproportionate to particle growth at 0.05 Cu^2^^+^ concentration is identified as an instrumental factor for the approximately 2.4-fold increase in strain observed in this particular sample, thereby driving the structural transition. Chemical compositional analysis corroborates the presence of a mixture of valence states and structural inhomogeneity alongside the substitution of Co^2^^+^ by Cu^2^^+^ at the B-site. DFT calculations indicate that the predominant interactions are AFM Co^2^^+^–O–Ru^4^^+^ interactions, with negligible contributions from Ru^5^^+^–O–Ru^5^^+^ interactions in the pristine sample.

The doping of Cu^2^^+^ introduces competing FM pathways (Cu^2^^+^–O–Ru^4^^+^/Cu^2^^+^), while AFM order remains evident at a doping level of 0.3 due to orbital asymmetry and strain-modulated exchange pathways. Measurements of magnetic susceptibility indicate an enhancement of the T_N_ to 39.8 K at a doping level of 0.05 Cu^2^^+^ from 28.7 K in the pristine sample, attributed to strain-induced octahedral distortions and the strengthening of AFM coupling, whereas at a higher doping concentration (0.3), disorder and competing FM interactions are reintroduced, resulting in a reduction of *T*_N_ to 25.2 K. A secondary transition observed at 19.2 K and the emergence of FM-like behavior above *T*_N_ imply the presence of magnetic inhomogeneity and local spin reorientation. CW analysis, bolstered by a finite-size-corrected model, substantiates the assertion that surface effects and strain influence the magnetic moments and Weiss constants, with larger particles exhibiting a resurgence of intrinsic magnetic behavior.

## Supplementary Material







## Data Availability

Data will be made available on request.

## Notes

X-ray crystallographic data of La_2_CoRuO_6_ monoclinic *P*2_1_ *c* (CIF)
X-ray crystallographic data of Sr_10_Cu­(RuO_5_)_4_ tetragonal *I*4*m* (CIF)
All-atom unit cells of pristine monoclinic (*P*2_1_/*c*) and Cu-doped tetragonal (*I*4/*m*) structures; crystallographic symmetry and atomic arrangement illustrations; La_2_CoRuO_6_ chemical identification and quantification; LCRO-5% chemical identification and quantification; LCRO-30% chemical identification and quantification (PDF)
