# Supplementary material for: B‑Site Cu2 + Substitution and Strain-Mediated Magnetic Evolution in La2CoRuO6 Double Perovskite: Insights from Experiment and DFT + U‑Corrected Calculations
Source: ACS Appl Mater Interfaces. 2025 Dec 15;17(51):69610–20. doi: 10.1021/acsami.5c18178 (PMC12754751; doi:10.1021/acsami.5c18178)
Supplement: Supplementary file 3 [file am5c18178_si_003.pdf]

Supporting Information

B-Site Cu^{2+} Substitution and Strain-Mediated Magnetic Evolution in La_2CoRuO_6 Double Perovskite: Insights from Experiment and DFT+U corrected calculations

Sibusiso Nqayi[1*] and Buyisiwe Sondezi[1]

[1]Rare Earth-Based Oxides and Nano Group, Department of Physics, University of Johannesburg, Cnr Kingsway Avenue and University Road, Auckland Park 2006, South Africa

**Corresponding Author*: sbunqayi@gmail.com

Supporting Information

Section S1: All-atom unit cells

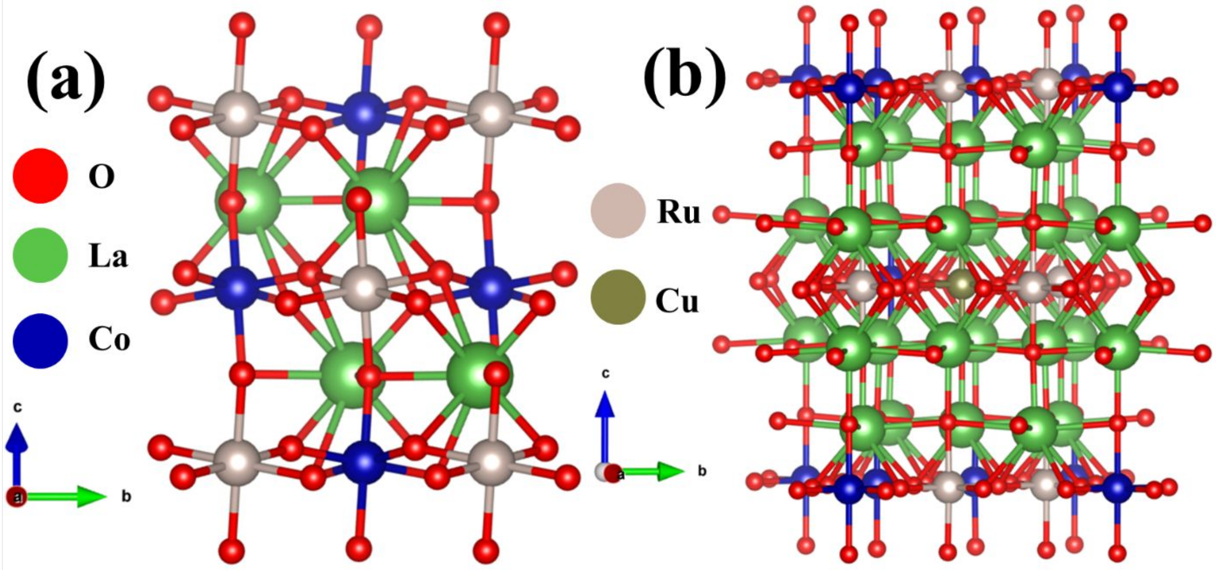

Figure S1. All-atom unit cells of (a) the pristine monoclinic structure ($P2_1/c$) and (b) the doped tetragonal structure (I4/m), illustrating the crystallographic symmetry and atomic arrangements corresponding to each phase.

Section S2: XPS surface chemical quantification with chemical ID and quantification.

Table S1. La_2CoRuO_6 Chemical ID and Quantification

Name	*Peak BE*	*FWHM eV*	*Atomic %*
O1s (Metal Oxide)	529.2	1.6	36.37
O1s (C-O)	531.5	1.6	34.77
O1s (C=O)	533.1	1.6	18.73
Co2p (Co2O3; Co3O4; CoOOH)	779.7	2.6	5.90
La3d (La2(CO3)3)	834.7	7.0	4.23

Table S2. LCRO 5% Chemical ID and Quantification

**Name**	**Peak BE**	**FWHM eV**	**Atomic %**
O1s (C-O)	531.4	1.7	39.49
O1s (Metal Oxide)	529.4	1.7	29.91
O1s (C=O)	533.0	1.7	17.27
Co2p (Co2O3; Co3O4; CoOOH)	779.9	3.3	5.89
La3d (La2(CO3)3)	835.2	6.9	4.33
Cu2p (Cu; CuO)	933.3	3.0	3.12

Table S3. LCRO 30% Chemical ID and Quantification

Name	*Peak BE*	*FWHM eV*	*Atomic %*
N1s (Nitride; Cyanides)	398.3	0.8	2.51
O1s (Metal Oxide)	529.5	1.7	27.79
O1s (C-O)	531.2	1.7	41.60
O1s (C=O)	532.8	1.7	12.98
Co2p (Co2O3; Co3O4; CoOOH)	780.2	3.8	5.99
La3d (La2(CO3)3)	835.1	3.7	5.16
Cu2p (Cu; CuO)	933.8	3.5	3.97
